# Decision-making approaches in transgender healthcare: conceptual analysis and ethical implications

**DOI:** 10.1007/s11019-021-10023-6

**Published:** 2021-05-18

**Authors:** Karl Gerritse, Laura A. Hartman, Marijke A. Bremmer, Baudewijntje P. C. Kreukels, Bert C. Molewijk

**Affiliations:** 1 Center of Expertise on Gender Dysphoria, Amsterdam UMC, location VUmc, de Boelelaan 1118, 1081 HZ Amsterdam, The Netherlands; 2 Department of Ethics, Law and Humanities, Amsterdam UMC, location VUmc, de Boelelaan 1089a, 1081 HV Amsterdam, The Netherlands; 3 Department of Psychiatry, Amsterdam UMC, Location VUmc, de Boelelaan 1118, 1081 HZ Amsterdam, The Netherlands; 4 The Netherlands Centre for Ethics and Health (CEG) and the Council of Public Health & Society (RVS) The Netherlands, Parnassusplein 5, 2511 VX Den Haag, The Netherlands; 5 Department of Medical Psychology, Amsterdam UMC, Location VUmc, de Boelelaan 1118, 1081 HZ Amsterdam, The Netherlands; 6 Faculty of Medicine, Center for Medical Ethics, Institute of Health and Society, University of Oslo, Kirkeveien 166, 0450 Oslo, Norway

## Abstract

Over the past decades, great strides have been made to professionalize and increase access to transgender medicine. As the (biomedical) evidence base grows and conceptualizations regarding gender dysphoria/gender incongruence evolve, so too do ideas regarding what constitutes *good* treatment and decision-making in transgender healthcare. Against this background, differing care models arose, including the ‘Standards of Care’ and the so-called ‘Informed Consent Model’. In these care models, ethical notions and principles such as ‘decision-making’ and ‘autonomy’ are often referred to, but left unsubstantiated. This not only transpires into the consultation room where stakeholders are confronted with many different ethical challenges in decision-making, but also hampers a more explicit discussion of what *good* decision-making in transgender medicine should be comprised of. The aim of this paper is to make explicit the conceptual and normative assumptions regarding decision-making and client autonomy underpinning the ‘Standards of Care’ and ‘Informed Consent Model’ currently used in transgender care. Furthermore, we illustrate how this elucidation aids in better understanding stakeholders’ ethical challenges related to decision-making. Our ethical analysis lays bare how distinct normative ambiguities in both care models influence decision-making in practice and how foregrounding one normative model for decision-making is no moral panacea. We suggest that the first steps towards *good* decision-making in gender-affirming medical care are the acknowledgement of its inherent normative and moral dimensions and a shared, dialogical approach towards the decision-making process.

## Introduction

Transgender (trans)^1^ persons often experience an incongruence between their gender identity and sex assigned at birth. In the past, this incongruence was taken to justify the need for psychiatric hospitalization and reparative psychotherapy (de Cuypere and Gijs 2014; Schwend 2020). Over the last decades, however, significant shifts in conceptualization and classification dovetailed with the emergence of more affirming care approaches (Beek et al. 2016; Schwend 2020). Currently, many trans people use hormones and/or seek surgery to express their experienced gender (Coleman et al. 2012).

The field of gender-affirming medicine is characterized by a growing, yet relatively slim (biomedical) evidence base, a great diversity of clients and treatment requests, as well as a complex history and multidisciplinary organization of care (Coleman et al. 2012). The field is unique compared to other parts of the Western healthcare system in that often a mental health professional determines eligibility for medical treatment, based on the assessment of an inherently subjective phenomenon that is currently classified as either a mental disorder (APA, 2013)^2^ or as a condition related to sexual health (WHO, 2018).^3^^,^ 4 These factors play into a state in which experts, clinicians and clients may disagree when it is appropriate to initiate medical treatment, how to organize decision-making and how to serve the client’s best interests. It is in this convoluted context that various professional organizations make available care models to outline guidelines for care, criteria for treatments and approaches to decision-making (Coleman et al. 2012; Reisner et al. 2015; Deutsch 2016; Hembree et al. 2017).

Currently, the most widely adopted care model is the Standards of Care 7 (SoC7) offered by the World Professional Association for Transgender Health (WPATH) (Coleman et al. 2012).^5^ Criteria for medical treatment consist of a minimum age; persistent, well-documented gender dysphoria; capacity to make a fully informed decision; and the absence of uncontrolled co-occurring medical or mental concerns. According to WPATH, mental health professionals are best prepared to diagnose and assess eligibility given their specific training and as medical treatment is intensive, often life-long and (partially) irreversible. This arguably makes their role into that of a ‘gatekeeper’; a role that is not without ethical challenges (Fraser and Knudson 2017; Schulz 2018). Clients argue ‘gatekeeping’ interferes with ‘autonomous’ decision-making (Bockting et al. 2010; Eyssel et al. 2017), while mental health professionals note the strain it puts on the therapeutic relationship, decision-making process and consequently their ability to offer *good* care (Lev 2009; Dewey 2013).

Seeking to foster ‘client autonomy’, a number of community health centres developed an approach to decision-making that has become known as the ‘Informed Consent Model’ (ICM) for transgender healthcare (Reisner et al. 2015; Deutsch 2016; Wylie et al. 2016; Schulz 2018).^6^ In the ICM, clients may access medical interventions—particularly hormone treatment—without mental health evaluation, and in some cases without a formal diagnosis (Schulz 2018). The precedence attributed to self-determination curtails the role of the health professional to providing information about risks and (side) effects of treatment, and obtaining informed consent (Schulz 2018). Here too, however, ethical challenges arise. For example: in the absence of a formal diagnosis and assessment of eligibility, how should the initiation of medical treatment be decided on (Giordano 2012)? Is this decision to be based on self-determination, or, for example, alleviation of gendered distress?

The SoC7 and ICM appear to differ greatly with respect to how decision-making and client autonomy are conceptualized and normatively laden. In both care models, however, this remains largely implicit. For example, the SoC7 speak of “[assisting] clients with making fully informed decisions” (Coleman et al. 2012, p. 181), and the ICM stresses the value of ‘client autonomy’ (Reisner et al. 2015). The specific interpretation and normative implications of these notions remain ambiguous: When is a decision *fully* informed? What interpretation of ‘client autonomy’ is intended and how should clinicians do justice to it? These ambiguities not only feed into clinical ethical challenges related to decision-making (Dewey 2013; Gerritse et al. 2018), but also impede a more explicit dialogue regarding what *good* decision-making in transgender healthcare is, or should be comprised of.

The aim of this paper is to provide an analysis of decision-making and client autonomy in two care models for adult transgender healthcare by means of a conceptual framework of decision-making models and corresponding notions of client autonomy. Explicating the conceptual and normative assumptions regarding decision-making and client autonomy in the SoC7 and ICM will aid in better understanding clinical ethical challenges related to these care models. This conceptual analysis has ethical implications for the discussion regarding what *good* decision-making in transgender healthcare should entail. Taken together, the ultimate aim of this paper is to elucidate and ameliorate current decision-making practices.

This paper is structured as follows: First, drawing from conceptual and ethical literature, we provide a theoretical background of decision-making models and corresponding notions of client autonomy. Then, for both SoC7 and ICM, we describe the specific decision-making processes regarding gender-affirming medical treatments. Next, we elucidate what decision-making model and notion of client autonomy are assumed in these models. Finally, we illustrate how this elucidation aids in better appreciating ethical challenges experienced by stakeholders in practice. We wrap up this paper by offering ethical implications for decision-making in transgender healthcare.

## Theoretical background: decision-making and client autonomy

In this section, we discuss *informative, paternalistic,* and *deliberative* decision-making models and corresponding interpretations of the principle of autonomy (Emanuel and Emanuel 1992). Decision-making models such as these build on previous work about the client–clinician relationship (e.g., Szasz and Hollander 1956) and inspire current debates surrounding shared decision-making (e.g., Elwyn et al. 2012; 2016; Stiggelbout et al. 2004, 2012) and person-centred care (e.g., Epstein and Street 2011; McCormack and McCance 2017). For our theoretical background, we draw from Emanuel and Emanuel (1992) as these authors explicitly link decision-making models to notions of client autonomy. Next to the conceptual clarity their work offers, their decision-making models remain central to the fields of clinical and bio-ethics as well as medical decision-making.

In the discussion of decision-making models and corresponding notions of client autonomy, the distinction between positive and negative freedom is helpful (Berlin 1969). Negative freedom refers to the absence of barriers, obstacles, or constraints by others (i.e., freedom *from*). Notions of ‘autonomy as negative freedom’ aim to protect the agent from interference by others. Conversely, positive freedom seeks to enhance the agent’s freedom to act and choose (i.e. freedom *to*). Interpretations of ‘autonomy as positive freedom’ thus emphasize and seek to support the capacity of a person to be the authentic source of their decisions and to live life according to one’s values, goals and plans (Berlin 1969).

### The informative model and a liberal legal ideal of autonomy

In the *informative* model, the obligation of the clinician is to provide information (e.g. on disease state, benefits and harms of treatment), so the client can select treatment that is most in line with her values. The clinician, in turn, executes the treatment selected by the client: ‘your wish is my command’ (Emanuel and Emanuel 1992). This model holds that clients are cognizant of their values and need relevant (medical) facts to make a decision that is right for them. As such, the informative model leaves little space for the values of clinicians or their interpretation of those of their clients. In the informative model, the clinician is thus conceived of as a technical expert, while the client is rendered a consumer whose autonomy is understood as choice of, and control over, decision-making (Emanuel and Emanuel 1992).

The informative model is underpinned by a ‘liberal legal’ notion of autonomy in line with Berlin’s negative concept of freedom (Berlin 1969). This interpretation of autonomy is also at the root of the (judiciary) doctrine of ‘informed consent’ (Beauchamp and Childress 2013) and often intended when autonomy is left unspecified or spoken of colloquially (Stiggelbout et al. 2004). A liberal legal notion of autonomy protects clients from undue interference by others in the decision-making process by demanding respect for the client’s integrity. Respecting autonomy in the context of an informative decision-making model implies a ‘negative obligation’ on part of the clinician to not constrain the client’s decision-making process (Beauchamp and Childress 2013). It is thus directed at those treating the client, rather than the client herself.

### The paternalistic model and autonomy as critical reflection

In the *paternalistic* decision-making model, the clinician tests and determines the client’s condition, and subsequently decides what treatment is most likely to promote health and well-being. This model presumes that there are shared, objective criteria for what is best, and that a clinician, based on experience or professional status, may discern what is in the client’s best interest (Emanuel and Emanuel 1992). Emanuel and Emanuel write about paternalism in the context of the client–clinician *relationship*. In the ethics literature, a further distinction is often made between weak and strong, or hard and soft, paternalistic *decision-making. *Beauchamp and Childress, for example, define paternalism as: “the intentional overriding of one person’s known preferences or actions by another, where the person who overrides justifies the action by the goal of benefitting or avoiding harm to the person whose preferences or actions are overridden” (2001, p. 178). According to these authors, weak paternalism refers to a situation where a clinician overrides—based on beneficence or non-maleficence—substantially non-autonomous actions, for example when a decision is not adequately informed, deliberated, or voluntary (Beauchamp and Childress 2013). Strong paternalism, on the other hand, involves superseding a substantially autonomous client.

Strong paternalism usurps client autonomy and is usually warranted by values such as protection (Beauchamp & Childress 2013). Weak paternalism, instead, is arguably grounded on a positive interpretation of autonomy closely related to the notion of ‘autonomy as critical reflection’ put forward in Dworkin’s 1988 theory of procedural independence. This notion of autonomy centers on the agent’s ability to critically reflect upon one’s (pre-reflexive) ‘first-order’ needs, wishes and desires, and to align them to values and preferences of a higher order. Acts and decisions are deemed autonomous when an agent can critically reflect on and identify with the influences that motivates them. Conversely, impulsive or pre-reflexive acts and decisions are regarded as non-autonomous (Dworkin 1988). In medical decision-making, this notion of autonomy asks of the clinician to assess whether a client’s decision is based on critical reflection and to what extent superseding is warranted.

### The deliberative model and relational autonomy

In the *deliberative* model, the clinician seeks to aid the client in determining what their health-related values are (Emanuel and Emanuel 1992). This requires the clinician to not only share information relevant to the client’s clinical situation but also to help elucidate what values correspond to which treatment option. The client may then come to choose a particular treatment in a more reflective manner. The clinician’s objectives may be more normative and include proposing why particular health-related values are more worthy to aspire to. Through moral deliberation and dialogue, the clinician and client work out what treatment option is best. In this process, the clinician not only lays out the different possibilities but, while avoiding coercion, also suggests what is in the client’s best interest based on deliberation.

Deliberative decision-making could thus be said to be underpinned by a positive, ‘relational’ notion of client autonomy which questions the idea of self-sufficiency and independence as principal moral values (Elwyn et al. 2012). In care ethics, these latter values are deemed perilous as they obscure values such as trust, caring and responsibility (Verkerk 2001; Mackenzie 2008). Care ethicists emphasize the need of having relationships in order to see oneself as autonomous, rendering autonomy a notion which cannot be appreciated nor developed in isolation from interpersonal relationships and social communication (Verkerk 2001). In a clinical context, ‘relational autonomy’ stresses the clinician’s positive obligation to take seriously individual differences to arrive at “genuine respect” (Mackenzie 2008, p. 530). Respecting autonomy in relational terms requires the clinician to actively attune to the client and their needs. In decision-making, this process involves a consideration of thoughts, values and corresponding treatment options through deliberation. It is reciprocal in that through an intention to mutual understanding, both client and clinician may arrive at new insights and revised values (Verkerk 2001).

With this theoretical background on decision-making and autonomy in place, we now turn to two care models for transgender healthcare.

## Care models in transgender healthcare: description and ethical analysis

In this section, we describe the decision-making processes regarding gender-affirming medical interventions in the SoC7 and ICM and elucidate what decision-making and notion of client autonomy these care models assume.

### Decision-making in the SoC7

WPATH has put forward their SoC since the end of the 1970s,^7^ when its goal was to protect those seeking gender affirming care as well as the reputation of providers (Fraser and Knudson 2017; Wyle et al. 2016). The SoC has since become the leading care model for gender-affirming care. Currently in its seventh iteration, the SoC7’s overall goal is “to provide clinical guidance for health professionals to assist transsexual, transgender and gender-nonconforming people with safe and effective pathways to achieving lasting personal comfort with their gendered selves, in order to maximize their overall health, psychological well-being and self-fulfilment” (Coleman et al. 2012, p. 165). In order to alleviate gender-related distress, the SoC7 outline options for (medical) care offered by a wide range of mental and somatic health professionals. The SoC7 are purposefully flexible^8^ to meet a range of transgender healthcare needs. While offering more leniency than preceding versions (cf. Meyer et al. 2002; de Cuypere and Gijs 2014), the current SoC7 retains a strong emphasis on the need for a comprehensive (mental health) assessment before clients may access gender-affirming medical treatments. Hence, the SoC7 foregrounds the importance of the role of a mental health professional in the decision-making process. The eligibility criteria for hormonal therapy and surgical treatment options are as follows (adapted from Coleman et al. 2012):

Criteria for Hormone Therapy, Breast/Chest Surgeries (1 Referral):^9^

1. Persistent, well-documented gender dysphoria
2. Capacity to make a fully informed decision and to give consent for treatment
3. Age of majority in a given country
4. If significant medical/mental concerns are present, they must be reasonably well controlled
  Criteria for Genital Surgery: Hysterectomy and Orchiectomy (2 Referrals):^10^
5. 12 continuous months of hormone therapy as appropriate to the patient’s gender goals (unless hormones are not clinically indicated for the individual)
  Criteria for Genital Surgery: Phalloplasty and Vaginoplasty (2 Referrals):^11^
6. 12 continuous months of living in a gender role that is congruent with their gender identity

Medical decision-making in the current SoC7 is described in tandem with clinicians’ tasks related to assessment and referrals, which include to: (1) assess gender dysphoria; (2) provide information regarding gender identity and expression and possible medical interventions; (3) assess, diagnose, and discuss treatment options for coexistent mental health concerns;^12^ (4) if applicable, assess eligibility, prepare, and refer for hormone therapy;^13^ and/or (5) surgery. Hence, the SoC7 suggest that the decision-making process not only involves informing clients about the range of gender identities, expressions and corresponding treatment options but also the need to evaluate eligibility through the assessment of gender dysphoria, psychosocial adjustment, and potentially coexisting mental health concerns.

WPATH’s SoC7 holds that mental health professionals are best suited to conduct these tasks given medical treatment is intensive, often life-long and (partly) irreversible. The role of mental health professionals in assessing gender dysphoria in the context of a client’s psychosocial adjustment “includes making sure that the gender dysphoria is not secondary to, or better accounted for, by other diagnoses” (p. 180). Another rationale for emphasizing the role of the mental health professional in decision-making is that clients presenting with gender dysphoria may struggle with a range of co-existing mental health concerns such as “anxiety, depression, self-harm, a history of abuse and neglect, compulsivity, substance abuse, sexual concerns, personality disorders, eating disorders, psychotic disorders and autistic spectrum disorders” which may be related or unrelated to gender dysphoria and/or a long history of minority stress (p. 180–181). Although the presence of mental health concerns does not necessarily preclude access to treatment, “these concerns need to be optimally managed prior to, or concurrent with, treatment of gender dysphoria [and] [i]n addition, clients should be assessed for their ability to provide educated and informed consent for medical treatments” (p. 181). According to the SoC7, “[a]dressing these [mental health] concerns can greatly facilitate the resolution of gender dysphoria, possible changes in gender role, the making of informed decisions about medical interventions, and improvements in quality of life” (p. 181).

In conjunction with the assessment of eligibility criteria, SoC7 calls on professionals to explore clients’ needs based on their goals for gender expression: “mental health professionals may facilitate a process in which clients explore these various options, with the goals of finding a comfortable gender role and expression and becoming prepared to make a fully informed decision about available medical interventions, if needed” (p. 180). To best facilitate the decision-making process, “mental health professionals need to have functioning working relationships with their clients and sufficient information about them” (p. 180). The SoC7 emphasize that treatment decisions are first and foremost a client’s decision. At the same time, mental health professionals “have the responsibility to encourage, guide and assist clients in making fully informed decisions and becoming adequately prepared”, sharing “the ethical and legal responsibility for that decision with the physician who provides the service” (p. 181–182).

### SoC7: Conceptual analysis of decision-making and client autonomy

Decision-making in the SoC7 appears to be characterized by both (weak) paternalistic and deliberative components and corresponding notions of client autonomy.^14^

First, the SoC7 emphasize the role of mental health professionals in evaluating gender dysphoria, checking for co-occurring concerns and assessing the client’s capacity to consent to medical procedures (Coleman et al. 2012). This highlights the clinician’s assessorial role in decision-making. Paternalistic decision-making is typified by a mechanism in which the clinician establishes the client’s condition and decides what treatment—if any—is in the client’s best interest (Emanuel and Emanuel 1992). Seeing how a client’s ability to engage in the decision-making process is contingent on the (mental) health professional’s assessment of eligibility criteria, decision-making in the SoC7 can be rendered weak paternalistic.

These weak paternalistic elements are indicative of a carefulness and vigilance that appear to be justified by the need to exclude those who are unable to cope with or might regret (irreversible) medical treatment; put briefly: to avoid harm. For example, the SoC7 speak of “cases … of people who received hormone therapy and genital surgery [who] later regretted their inability to parent genetically related children” (p. 196). Similarly, the SoC7 stress the need to screen for coexisting mental concerns as these might better account for the client’s gender dysphoria or hamper her ability to give informed consent (Coleman et al. 2012). These fragments imply that clients’ (gendered) values might be inchoate or erroneous and presume a notion of ‘autonomy as critical reflection’ (Dworkin 1988). Indeed, in the process of assessing eligibility, mental health professionals appear to ascertain whether a client’s wish for medical treatment is based on critical reflection in order to foster beneficence and non-maleficence. This corresponds with empirical-ethical findings in clinical practice: in ‘complex’ cases where doubt arises regarding the ‘authenticity’ of gender dysphoria or the client’s ability to give *full* informed consent, (mental) health professionals may abstain from granting access to medical treatment based on values such as protection (Gerritse et al. 2018).

Meanwhile, however, the SoC7 stress a more deliberative side to decision-making in the more general task bestowed on mental health professionals to work out, together with their clients, what treatment options fit best with their values. The SoC7 press mental health professionals to recognize that treatment decisions are “first and foremost a client’s decision” (p. 181). In reaching those decisions, mental health professionals are expected to “encourage, guide and assist clients with making fully informed decisions” necessitating “functioning working relationships with their clients and sufficient information about them” (p. 181). In fact,

an important task of mental health professionals is to educate clients regarding the diversity of gender identities and expressions and the various options available to alleviate gender dysphoria. Mental health professionals then may facilitate a process in which clients explore these various options, with the goals of finding a comfortable gender role and expression and becoming prepared to make a fully informed decision about available medical interventions, if needed. (p. 180)

These latter quotes contrast with paternalistic conceptions of decision-making and imply a process more in line with the deliberative model where clinician and client, collaboratively and through moral deliberation work out what treatment option is best (Emanuel and Emanuel 1992).

These deliberative elements of decision-making in the SoC7 hint at the presupposition of a more relational conception of client autonomy (Mackenzie 2008). Indeed, the SoC7 stress the importance of assisting clients in making informed choices, as well as individualizing and working out together with clients a treatment plan that aligns with their experienced gender identity. This emphasis can be read as a positive obligation on the part of the clinician to take differences between clients seriously and attune to the needs of the care-receiver, which necessitates a mutual engagement to the deliberative process of care (Verkerk 2001).

Thus, decision-making in the SoC7 appears to be normatively underpinned by weak paternalistic as well as deliberative assumptions. Correspondingly, client autonomy is implicitly conceptualized both as ‘critical reflection’ and relationally. It appears that the weak paternalistic duties of the mental healthcare professional are informed by values such as ‘non-maleficence’ and ‘protection’ with corresponding norms such as ‘regret or damage should be prevented’. The deliberative duties, on the other hand, could be underpinned by values such as ‘self-actualization’, or ‘happiness’ and related to norms such as: ‘people should be able to realize their gendered selves’.^15^ These values, convictions and norms, however, remain largely implicit and unspoken in the SoC7. In the next section, we illustrate how this elucidation aids in better understanding ethical challenges encountered by stakeholders.

### SoC7: Clarifying clinical ethical challenges in decision-making

The discussion regarding decision-making in the SoC7 centers particularly on what has become known as ‘gatekeeping’: the state of affairs in which a mental health professional assesses eligibility and in effects holds final decision-making power over medical treatments (Dewey, 2013; Fraser & Knudson, 2017; Lev, 2009). As mentioned above, mental health professionals in SoC7 are expected to perform both assessorial as well as more supportive tasks in the process of decision-making. These assessorial and supportive tasks are rooted in diverging normative assumptions regarding decision-making and conceptualizations of client autonomy, and are hence morally conflicting. Especially when bestowed on one single mental health professional, it is unsurprising that these tasks may give rise to moral challenges on both sides of the dyad.

A recent German survey shows how a great majority of trans clients consider mental health counseling to be helpful during their medical transition, both for trans-related and unrelated issues (Eyssel et al. 2017). Mandatory consultation with a mental health professional in the context of decision-making on gender-affirming medical care, on the other hand, can be experienced as pathologizing (Murphy 2016) or an undue burden (Cavanaugh et al. 2016). Indeed, some trans clients report that they run up against barriers in accessing medical care (Safer et al. 2016), or feel medical decisions are unnecessarily prolonged (Eyssel et al. 2017). In effect, some clients are reluctant to engage with their mental health professional, feel that they have to ‘jump through hoops’ or cannot tell the ‘full story’ (Cavanaugh et al. 2016; Eyssel et al. 2017; Fraser and Knudson 2017; Schulz 2018). This makes for a clinical encounter in which clients may feel hampered to freely share information regarding their gender dysphoria/gender incongruence or potentially coexisting concerns (Benson 2013; Budge and Dickey 2017; Fraser & Knudson 2017). Clients’ key ethical questions may be phrased as follows: In the process of decision-making, should I perceive my clinician as a guardian, friend, or someone with shifting professional roles and values? What are my clinician’s assumptions regarding my autonomy, capacity, and moral status? Fundamentally, should I trust my clinician? Consequently, clients may arrive as educated social actors who know how they should frame their narrative of gender identity, development and dysphoria/incongruence to convince their mental health professional during the decision-making process (Cavanaugh et al. 2016; Budge and Dickey 2017; Schulz 2018). As a consequence, another explicit ethical question of the client could be: should I frame my (gendered) narrative authentically, or so as to convince my clinician that my appeal for treatment should be respected?

Mental health professionals, on their part, have questioned how their professional role and responsibility should materialize in decision-making. Indeed, some note that they find their professional responsibility ambiguous, and their various roles conflicting (Gerritse et al. 2018). Quoting a mental health professional regarding the decision to start hormone therapy: “[T]o what extent should it be our responsibility to decide regarding these risks? Is that one’s role as a caretaker, doctor, or psychologist (Gerritse et al. 2018, p. 2327)? This could indicate that this clinician is faced with ethical questions such as: What should be my (professional) role and responsibility in this decision-making process? How should I consolidate my paternalistic duties with those that are more deliberative? How should I navigate these differing tasks to build the trust and rapport necessary for *good* care and decision-making?

Furthermore, mental health professionals are often aware of how their role conflict impacts client’s presentation. For example, regarding a persistent, life-long and ‘early-onset’ history of gender dysphoria, a Dutch psychologist noted in a case discussion how “maybe this story is being told, because [the client] thinks it increases their chance of getting treatment” (Gerritse et al. 2018, p. 2327). Similarly, a U.S. mental health professional experienced an ethical dilemma in having to rely on client narratives in decision-making, while knowing that clients may feel compelled to alter their histories (Dewey 2013). Again, when made more explicit, these clinicians could face ethical questions such as: Knowing that my assessorial tasks might preclude my client from talking freely, how should my client and I arrive at a sensible treatment decision? Besides hampering client–clinician rapport building, another perilous implication of selective information exchange in the decision-making process is that potentially important information, preferences and values are omitted from deliberation and final decision-making (Dewey 2013; Schulz 2018). When unable to hear the authentic client narrative, both mental health professional and client may be hampered in arriving at a *good* treatment decision.

Having expounded the SoC7 by means of our theoretical background, we now turn to the ICM.

### Decision-making in the ICM

In an alternative care model based on the principle of informed consent, transgender clients may access gender-affirming treatment by directly engaging with a primary care provider. This ‘informed consent model’ for (particularly hormonal) gender-affirming medical care is implemented in several community health centers in the United States and Canada (Deutsch 2012, 2016; Cavanaugh et al. 2015; Reisner et al. 2015; Callen Lorde Community Health Center 2018). The ‘ICM’ is in fact an umbrella term for a variety of closely-related care approaches in gender-affirming medical care that focus on informed consent as a means to structure decision-making: while the SoC also have informed consent procedures put in place, “the focus [in the ICM] is on obtaining informed consent as the threshold for the initiation of hormone therapy in a multidisciplinary, harm-reduction environment” (Coleman et al. 2012, p. 188).^16^ The main difference between the SoC7 and ICM is that “the SoC puts greater emphasis on the important role that the mental health professional may play in alleviating gender dysphoria and facilitating changes in gender role and psychosocial adjustment” (ibid).

For example, in 2007, Fenway Health in Boston implemented their interpretation of an ICM for gender-affirming hormone therapy. Their aim was to remove barriers such as a mental health evaluation and the necessity of psychotherapy or ‘real life experience’^17^ that were called for in the sixth version of the SoC that was in force at the time (Reisner et al. 2015).^18^ Their interpretation of the ICM stipulates that clients may access hormone therapy after engaging in a ‘hormone readiness assessment’. Their eligibility criteria for gender-affirming hormone therapy are:

- 1. Candidates for hormone therapy must be 18 years old and able to make and give informed consent for therapy.
- 2. Candidates have a consistent and persistent gender variant identity meeting the DSM-5 criteria for gender dysphoria. If significant mental or medical health conditions are present, they must be reasonably well controlled.

The initial assessment is usually made by a primary care provider who is competent in (1) establishing the client’s readiness and appropriateness through a bio-psycho-social screening; (2) engaging in and assessing the informed consent procedure, as well as (3) prescribing hormone therapy and (4) providing follow-up care (Cavanaugh et al. 2015). The initial assessment generally requires two to three appointments, and hormone therapy is often initiated in that timespan as the result of a collaborative decision between client and clinician. Along with a medical evaluation, the assessment includes the clinician taking the history of a client’s realization and understanding of gender identity and assessment of the presence of gender dysphoria (Cavanaugh et al. 2015). This includes a discussion of treatment goals and options, as well as exploring and assessing a client’s social and mental health history. As hormone treatment is expected to be life-changing and partially irreversible, informed consent procedures are put in place. They include the provision of information regarding the benefits, risks, usage and expected time-course, and discussion of “realistic expectation of changes” and, amongst others, effects on fertility (Cavanaugh et al. 2015, p. 7).

Although Fenway Health’s interpretation of the ICM draws from the DSM-5 diagnostic criteria for Gender Dysphoria, psychotherapy and evaluation by a mental health professional are not requirements to access hormone therapy.^19^ However, their interpretation of the ICM does not categorically exclude the involvement of mental health professionals. More specifically, supportive mental health is distinguished from gender-evaluating assessments and the process of evaluating informed consent (Cavanaugh et al. 2016). When the primary care provider doubts the client’s ability to give valid informed consent or suspects the presence of a mental health condition that is not “reasonably well controlled”, a client may be referred to a mental health professional (Cavanaugh et al. 2015, p. 8). The latter, in turn, works together with the primary care provider and client to establish readiness for hormone and surgical treatment. In a supportive role, conversely, mental health professionals are encouraged to support a balanced and optimally healthy gender affirmation process (Cavanaugh et al. 2015). As such, Fenway Health’s ICM stresses the potential importance of starting or continuing psychotherapy or mental health treatment during (medical) transitioning (Cavanaugh et al. 2015).

### ICM: Conceptual analysis of decision-making and client autonomy

Decision-making in the ICM, compared to the SoC7, takes an alternate starting point in that the mental health professional often does not play an explicit role. In the ICM, after clinicians provide information regarding treatment and conduct an initial bio-psycho-social screening, clients are asked to provide informed consent. Indeed, “the focus is on obtaining informed consent as the threshold for the initiation of hormone therapy” (Coleman et al. 2012, p. 188). As the name suggests, decision-making in the ICM shows evident parallels to the *informative* decision-making model (Emanuel and Emanuel 1992). In this relationship, the client receives all relevant medical information from the clinician to make a decision that aligns with her values. Correspondingly, the role of the client is that of a well-informed consumer, while the clinician is a technical expert whose values should not interfere in the decision-making process. In the words of the Callen Lorde Community Health Center (2018, p.3), one of the pioneering clinics advocating the ICM:

We strive to establish relationships with patients in which they are the primary decision makers about their care, and we serve as their partners in promoting health. This partnership supports the patient's ongoing understanding of the risks and benefits of hormone therapy. By providing thorough education around hormones and general health, we also aim to enhance a patient's ability to make informed decisions about all aspects of their health.

At the root of the principle of informed consent is a liberal legal ideal of autonomy (Stiggelbout et al. 2004). Indeed, in the ICM, this notion of autonomy as negative freedom is upheld and respected through minimizing undue external interference on the client’s decision-making process. It stipulates that when a client can give valid informed consent, they ought to be able to choose ‘freely’, i.e., with as little intrusion as possible: “We believe patients who are well informed have a right to make their own decisions. (Callen Lorde Community Health Center 2018, p 3).

There are, however, differences between various renditions of the ICM. Fenway Health’s interpretation, for example, appears to concurrently prescribe weak paternalistic duties in decision-making through the assessment of Gender Dysphoria, the client’s mental health status, and capacity to give informed consent. When in doubt, a clinician may refer the client to a mental health professional for further evaluation. What thus remains ambiguous is [when] and to what extent decision-making in the ICM is concurrently underpinned by a positive notion of ‘autonomy as critical reflection’ (Dworkin 1988).

### ICM: Clarifying clinical ethical challenges in decision-making

Clinical ethical challenges in decision-making in the ICM can be schematically subdivided in those pertaining to the limits, or ambiguity of ‘informed consent’.

First, emphasizing (especially a limited, or legalistic take on) ‘informed consent’ in decision making could corrode the therapeutic alliance (Fraser and Knudson 2017). For example, when informed consent becomes the threshold for decision-making, how should we do justice to the ‘expert status’ of the clinician? Here, a clinician could ask: Should an informative decision-making model preclude me from making my (professional) knowledge, experiences and values explicit? In the process of decision-making, should I keep my values, apprehensions and clinical judgement regarding potential outcomes at bay, or instead deliberate on them with my client? Likewise, an informative decision-making model might not meet the needs and concerns some clients have surrounding gender-affirming medical care. For example, those with uncertain (gendered) values could be underserved by a decision-making process resting on informed consent and a negative, liberal legal notion of their autonomy.

Relatedly, the notion that a mental health screening may be bypassed in decision-making for medical treatment prompts ethical questions. Given the (partial) irreversibility^20^ of medical treatments, the phenomenology of gender incongruence/gender dysphoria which is often characterized by a history of distress, and the high prevalence of coexisting mental health concerns, some argue that doing away with (mandatory) assessment of a mental health professional is unwarranted. Selvaggi and Giordano (2014), writing on gender-affirming surgeries that are also offered in other care contexts without mandatory mental health screening, argue that “offering or requesting psychological assistance is in no way … an attack to the patient’s [*sic*] autonomy” (p. 1177). Echoing a positive, care-ethical interpretation of autonomy, they add: “To treat people as equal does not mean that they should be treated in the same way but with the same concern and respect, so that their unique needs and goals can be achieved (p. 1177). Taken together, a clinician’s ethical question here could be formulated as: Does the procedure of informed consent sufficiently support my client’s autonomy in decision-making?

Second, in the absence of clear criteria, the ambiguity of ‘informed consent’ could give rise to a state in which the assessment of clients’ ability to give *valid* or *full* informed consent becomes a displacement for ‘gatekeeping’ (Dewey 2013). A clinician assessing informed consent in the ICM must ensure that their client understands the nature of the interventions, consequences, risks and benefits as well as alternatives to medical interventions (Cavanaugh et al. 2015; Murphy 2016; Schulz 2018). It remains unclear, however, when and how this understanding is sufficient, but also when a clinician should be considered adequately and appropriately trained to assess this. Indeed, a clinician could ask: What should count as *valid* or *full* informed consent to treatment? How should we go about assessing the impact of a co-occurring mental problem on the capacity to give informed consent? When is a referral to a mental health professional justified or obligatory?

Relatedly, an ethical concern is that the purported positive impact on the client–clinician relationship envisioned by advocates of the ICM may prove fruitless. For example, the emphasis put on the assessment of client capacity to make a *fully* informed decision might give rise to the reproduction of a gatekeeping dynamic hinging on the assessment of decisional capacity. In fact, a qualitative interview study illustrates how clinicians in transgender healthcare often use the rhetoric of informed consent, while failing to put it to practice: “In performing informed consent, providers revert to a paternalistic model of care, which amplifies their medical authority while veiling power differentials in their clinical encounters and decision-making in trans medicine” (shuster 2019, p. 190). This quote demonstrates how the ambiguity of informed consent may obscure an underlying notion of client autonomy as critical reflection (Dworkin 1988).

## Discussion

In this paper, we elucidated conceptual and normative assumptions regarding decision-making and client autonomy in two care models for transgender healthcare: the SoC7 and ICM. Subsequently, we illustrated how these assumptions feed into distinct role and value conflicts influencing decision-making in practice.

In the SoC7, the tension between mental health professionals’ assessorial and supportive tasks indicates a tension between weak paternalistic and deliberative assumptions in decision-making and conflicting conceptions of client autonomy. This tension gives rise to a role conflict on part of the clinician which could be met with apprehension, mistrust and selective information exchange on part of the client. Frustrating the clinical partnership in effect, both mental health professional and client may be hindered in arriving at a properly deliberated and attuned medical decision, and ultimately in realizing *good* care. At first glance, the ICM appears to bypass this ethical predicament. However, upon closer inspection, our analysis suggests that the ICM is no ethical cure-all. Indeed, attuning to the individual needs of transgender clients and collaborative decision-making in the ICM could be hampered by an informative model rooted in a legalistic and narrow interpretation of informed consent and client autonomy. Moreover, the normative ambiguity of ‘informed consent’ and ‘client autonomy’ potentially veils professionals’ paternalistic duties undermining the ICM’s very project.

Our analysis thus reveals how decision-making in transgender healthcare is characterized by inherent moral and normative dimensions that often remain implicit. The notion that these moral and normative dimensions are *inherent* implies that stakeholders’ norms and values regarding decision-making will continue to differ and, in some cases, come into conflict. As a consequence, clinical ethical challenges are arguably inevitable. Our analysis thus suggests that seeking to ‘resolve’ clinical ethical challenges by emphasizing one ‘ideal’ care model for decision making is futile, and, moreover, potentially perilous as such reflexes tend to obscure the underlying moral and normative dimensions at the root of these challenges. We believe our analysis illustrates, instead, how explicating these moral and normative dimensions can aid in recognizing, better understanding, and handling (but not ‘resolving’) clinical ethical challenges regarding decision-making.

### Ethical implications

Based on above, we plea for more explicit attention to the (fluid and evolving) moral and normative dimensions of (shared) decision-making in transgender healthcare. It is increasingly recognized that clients do not always have strong, clear, or stable values they can issue after being sufficiently informed about a specific treatment modality which can then be relied on to secure *good* decision-making (Entwistle and Watt 2016). Indeed, clients’ opportunities to share decision-making can be contingent on their conditions, co-occurring mental and medical concerns, and socio-economic as well as cultural circumstances (Entwistle and Watt 2016; Gerritse et al. 2018). Clinicians in transgender healthcare, too, arrive in the consultation room with a diverse set of implicit normative presumptions and values regarding decision-making (Gerritse et al. 2018). Moreover, given the multidisciplinary and sequential character of transgender healthcare (Coleman et al. 2012), we can best understand decision-making as an ongoing process which is distributed across people, places and times, defying the archetypal client–clinician decision-making dyad. For example, values regarding (shared) decision-making may depend not only on the type of intervention (e.g. hormonal or surgical), the clinician’s multidisciplinary background, but also the particular gender identity, future treatment wishes and family background of the client (Gerritse et al. 2018; Richards et al. 2016).

These differing values, needs and complexities underscore the need to diversify and individualize decision-making models in transgender healthcare. To reason with Emanuel & Emanuel: “clearly, under different clinical circumstances, different [decision-making] models may be appropriate” (1992, p. 2225). We suggest that the first step towards this ongoing process of shaping *good* (shared) decision-making in transgender healthcare is making explicit stakeholders’ various normative assumptions, perspectives and preferences regarding (shared) decision-making. Indeed, the notion that there is no panacea when it comes to decision-making models in transgender medicine emphasizes the need for dialogue and transparency regarding what it means to co-construct a *good* decision-making process; before but also during the actual process of decision-making. This is in keeping with recent accounts stressing the role of dialogue and dialogical consensus as the moral basis for (shared) decision-making (Walker 2019; Walker and Lovat 2019). Future dialogical empirical-ethics research with and for all involved stakeholders could aid in explicating their normative assumptions and help to jointly develop a normative framework outlining what a *good* (shared) decision-making process in transgender medicine could entail (Hartman et al, 2019a; Widdershoven et al. 2009). Besides a normative framework, practical avenues to support stakeholders in dealing with moral challenges in the actual decision-making process are necessary. Dialogical empirical-ethics research can inform the co-creation of ethics support tools, such as a moral compass (Hartman et al, 2019b). Such ethics support tools may aid in methodically elucidating and reflecting on clients’ and clinicians’ values relevant to (shared) decision-making (see, e.g., Hartman et al. 2018). In this way, both empirical-ethics research and ethics support tools may not only contribute to reflecting on but also foster *good* decision-making in gender-affirming medical care.

### Limitations and further work

There are several limits to this paper. First, conceptually we treated the SoC7 and ICM as distinct care models whereas in practice a variety of local interpretations of both SoC7 and ICM are developed and used. Furthermore, these guidelines inform practice, but are not a direct reflection of what goes on *in* practice, since there will always be a level of discretion and interpretation, both individually and institutionally. Research into the similarities and differences between these local interpretations of the SoC7 and ICM, as well as ethical challenges encountered by stakeholders working with these various guidelines in practice is necessary to help further the discussion. This research could benefit from ethnographical and observational methods. Given the paucity of empirical literature on clinical ethical challenges in the decision-making context of the ICM, further research into this area is especially warranted.

Second, our analysis relied on literature on decision-making models (Emanuel and Emanuel 1992) and client autonomy (Stiggelbout et al. 2004). Although this particular literature is widely used and appropriate for the themes at hand, it by no means offered an exhaustive analysis of decision-making in gender-affirming medical care. Analyses drawing from other ethical theories, or frameworks such as the four principles approach (Beauchamp and Childress 2013) or care ethics (e.g., Tronto 1993) could offer insights that should be compared and contrasted to those presented here. Likewise, approaching the topic through the prisms of shared decision-making (e.g., Elwyn et al. 2012, 2016; Stiggelbout et al. 2004) and person-centred care (e.g., Epstein and Street 2011; McCormack and McCance 2017) could further the conversation.

Third, we did not explicitly take into account the broader normative context in which decision-making takes place. However, the broader normative context of transgender healthcare (including cultural, financial, legal, social dimensions as well as professional expert opinion) has a pervasive influence on the ways in which decision-making regarding gender-affirming medical care is organized, guidelines are developed and treatment is offered (see, e.g., Dewey 2013; Cavanaugh et al. 2016; Gerritse et al. 2018). Future research should acknowledge and further investigate the impact of this broader normative context on ethical challenges in (shared) decision-making.

Fourth, foregrounding the ethical dimensions of (shared) decision-making could obscure the presence and more explicit handling of more fundamental epistemological and ontological views and questions regarding gender dysphoria/gender incongruence, its treatment and its impact on (shared) decision-making. It remains ambiguous, for example, how gender dysphoria/gender incongruence is conceptualized in the SoC7 and ICM, by stakeholders in practice and how this influences decision-making. How do these ambiguities impact, for example, the idea and possibility of medical indication in the field of gender affirming medical care? Elucidating these questions through the philosophy of psychiatry (e.g., Kendler et al. 2011), medical anthropology (e.g., Mol 2002), or literature on medical indication (e.g., Jonson et al. 2015), could foster a deeper understanding of how epistemological and ontological ambiguities shape the normative framework in which (shared) decision-making in transgender medicine takes place.

## Conclusion

The discussion as to what comprises *good* decision-making in transgender healthcare is thorny and ongoing. In order to contribute to this discussion, we elucidated the normative assumptions regarding decision-making and client autonomy in two current care models: the SoC7 and ICM. For both models, we formulated ‘key’ ethical questions illustrating how our analysis aids in better appreciating ethical challenges of stakeholders in practice. More specifically, our ‘key’ questions lay bare how the inherent normative ambiguities regarding decision-making and client autonomy in both SoC7 and ICM could frustrate decision-making in practice. Our analysis suggests that the inherent moral and normative dimensions of decision-making in transgender healthcare entail that ethical challenges regarding what *good* decision-making entails are inevitable. Rather than devising or debating care models aiming to resolve these ethical questions and dilemmas, we argue that the first steps towards a *good* decision-making process in transgender healthcare are the acknowledgement of and dialogue about its inherent normative presumptions and values. In fact, that should be the ground on which *good* decision-making in gender affirming medical care is rooted.

## Footnotes

1 In this paper, we use ‘trans’ and ‘transgender’ interchangeably as umbrella terms referring to various forms of gender identities, roles and expressions that differ from those normatively expected of one’s sex assigned at birth.

2 The American Psychiatric Association (APA), in the fifth edition of the Diagnostic and Statistical Manual of Mental Disorders (DSM-5), has outlined criteria pertaining to the psychiatric diagnosis of Gender Dysphoria (capitalized) (APA, 2013).

3 The World Health Organization (WHO), in the eleventh edition of the International Classification of Diseases (ICD-11), moved the classification of Gender Incongruence out of the “Mental and behavioral disorders” chapter and into the new “Conditions related to sexual health” chapter (WHO, 2018).

4 Regarding this inconsistency, experts in the field write: “The diagnostic classifications of disorders related to (trans)gender identity is an area long characterized by a lack of knowledge, misconceptions and controversy. The placement of these categories has shifted over time within both the DSM and ICD, reflecting developing views about what to call these diagnoses, what they mean, and where to place them” (Drescher et al. 2012, p. 568).

5 In various (multidisciplinary) clinics, the SoC are implemented in local care models.

6 While acknowledging the myriad meanings and interpretations of informed consent (Beauchamp 2011), we focus here on how ‘informed consent’ is intended/operationalized in the specific context of the ICM for transgender healthcare.

7 From 1979 until 2007 WPATH was known as the Harry Benjamin International Gender Dysphoria Association.

8 The criteria for hormonal and surgical treatment options allow for motivated departures for a variety of individual, institutional, legal, or policy-related reasons (Coleman et al. 2012, p. 166).

9 In line with the sixth version of the SoC, surgical interventions currently require one (for breast/chest surgeries) or two (for genital surgeries) referral letters from mental health professionals (Coleman et al. 2012).

10 Including criteria 1 to 4 above.

11 Including criteria 1 to 5 above.

12 The purpose of a mental health assessment is to determine “gender identity and gender dysphoria, the impact of stigma attached to gender nonconformity on mental health, and the availability of support” (p. 180).

13 The SoC7 note that although mental health professionals are best prepared to conduct these tasks, medical professionals with appropriate training, i.e., primary care physicians, nurses, and nurse practitioners, may also carry them out.

14 Throughout the SoC7, ‘autonomy’ is not explicitly mentioned.

15 Previous empirical-ethics research identified some of these underlying values and norms guiding decision-making of clinicians working in a multidisciplinary clinic where a local interpretation of the SoC7 is used (Gerritse et al. 2018).

16 In this paper, we predominantly draw from the ICM model offered by Fenway Health in Boston (2015).

17 A period in which transgender clients were expected to life full-time in their experienced gender role.

18 In the current SoC7, WPATH notes that since the guidelines are flexible, “[ICM] protocols are consistent with … the Standards of Care, Version 7” (Coleman et al. 2012, p. 187).

19 Of the twelve sites working with the ICM included in a study by Deutsch in 2012, four required contact with a mental health provider prior to the initiation hormone treatment.

20 Some authors (e.g., Cavanaugh et al. 2016) have challenged the notion that hormone and even most surgical options should be considered irreversible.
